# Bleomycin-Induced Fulminant Hyperpyrexia: A Report of Two Cases and Review of Literature

**DOI:** 10.7759/cureus.29785

**Published:** 2022-09-30

**Authors:** Mussadique Ali Jhatial, Sameen Bin Naeem, Mansoor Abbas, Naqib Ullah Baloch, Syed W Bokhari, Bushra Ahsan, Usman Ahmad, Rizwan Masood Sheikh

**Affiliations:** 1 Medical Oncology, Shaukat Khanum Memorial Cancer Hospital and Research Centre, Lahore, PAK; 2 Medical Oncology, Shaukat Khanum Memoiral Cancer Hospital and Research Centre, Lahore, PAK

**Keywords:** fulminent, germ-cell tumor, lymphoma, hyperpyrexia, bleomycin

## Abstract

Bleomycin is a commonly used cytotoxic agent that has proven its efficacy over the years. Though a common part of many protocols targeting lymphomas and germ cell tumors, it does have some serious adverse effects. Bleomycin is notorious for pulmonary toxicity and very rarely may cause fulminant hyperpyrexia. We describe two cases of classical Hodgkin’s lymphoma (cHL) developing acute fulminant hyperpyrexia after administration of the first dose of bleomycin as part of chemotherapy protocol. This is a rare adverse reaction that closely mimics anaphylaxis and has an unpredictable and possibly fatal course. Health care professionals involved in the administration of chemotherapy need to be very vigilant in monitoring for symptoms of this reaction.

## Introduction

Bleomycin, a glycopeptide antibiotic [1], is used frequently as an anti-cancer drug against lymphoma and germ cell cancers [2]. It is metabolized by bleomycin hydrolase enzyme to inactive end-products [3]. Although it is safe and effective, frequent dose-limiting toxicity of bleomycin is pneumonitis leading to a five to six times higher risk of chronic pulmonary dysfunction with a restrictive deficit [4]. There is up to a 10% risk of bleomycin-induced pneumonitis (BIP) with up to 20% risk of BIP-related mortality [5]. Bleomycin hydrolase enzyme activity is postulated to be deficient in lungs leading to accumulation of the drug in lung tissue, subsequently leading to oxidative damage because of activation of alveolar macrophages [6-7]. Risk factors for BIP include older age, history of smoking, exposure to radiation, acute or chronic renal dysfunction, exposure to high flow oxygen, and cumulative dose of bleomycin of >400 units [8].

Apart from this serious side effect, bleomycin is usually tolerated well, with often a milder and self-limiting febrile reaction which commonly occurs shortly after its administration [9-10]. Rarely, bleomycin may lead to a potentially fatal, fulminant hyperpyrexia [3] that was first reported with high grade fever, followed by cardiorespiratory collapse and subsequently death subsequently in four cases of classical Hodgkin's lymphoma (cHL) [11]. Further cases have been reported with hyperpyrexia either during the first exposure or, more rarely during subsequent exposures [12-14]. Such fatal reactions have been reported between the years 1970 and 2000. However, since it is common practice to give a test dose prior to a full dose bleomycin injection, fulminant hyperpyrexia has been rarely observed in recent years except for a case reported by Suzuki et al. in 2010 [15], and recently in three cases reported by Bond et al. in 2018 [11]. Though the rarity of this syndrome precludes the understanding of underlying pathophysiology, the release of pyrogenic cytokines leading to a systemic inflammatory syndrome similar to cytokine release syndrome (CRS) has been postulated to be the etiopathogenesis of this life threatening event [11]. Pre-existing fever and systemic inflammatory response syndrome have been associated with almost all the cases reported in literature.

## Case presentation

We report two cases of bleomycin-induced fulminant hyperpyrexia (BIFH) in patients with cHL that developed within 20 minutes of receiving the drug.

Patient one

**Figure 1:**
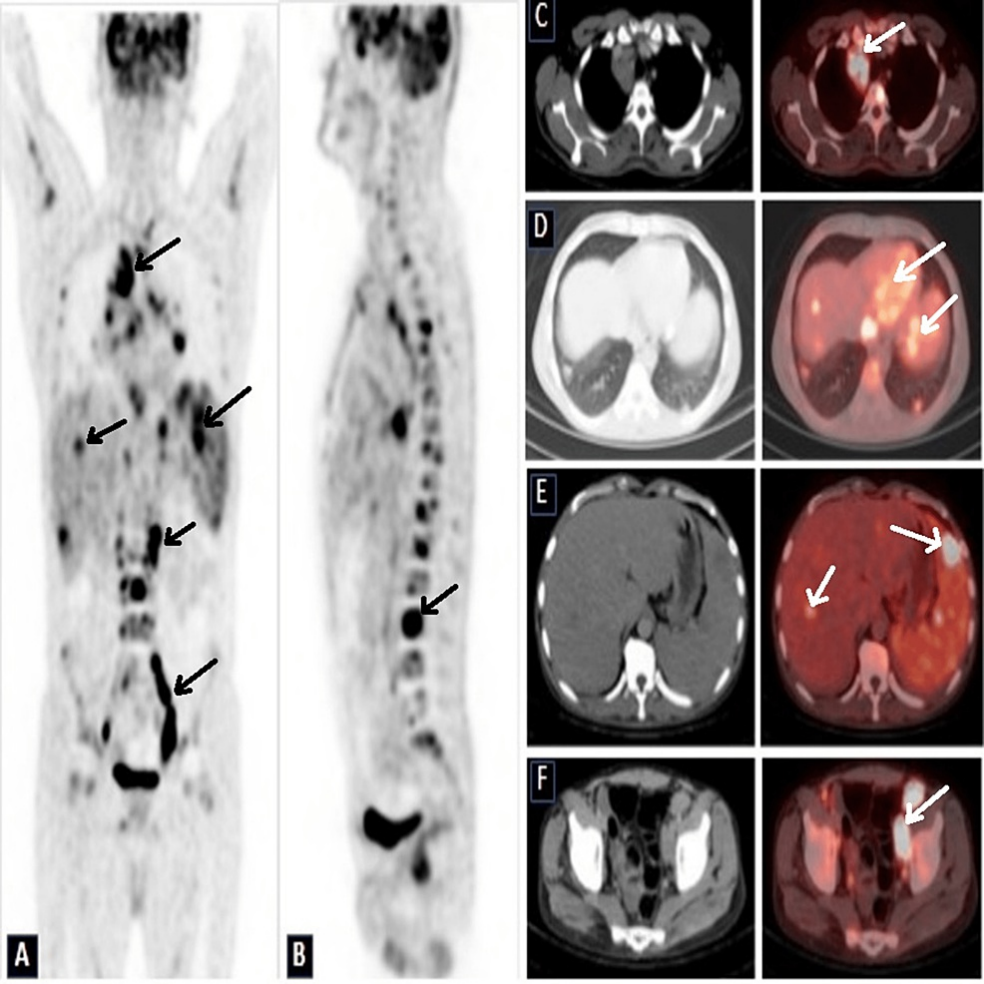
Coronal (A) and sagittal (B) maximum intensity projection (MIP) views of baseline F18-FDG PET-CT showing hypermetabolic nodal disease above and below the diaphragm with hepatosplenic and marrow involvement. C: Axial CT and fusion PET-CT images at different levels show F18-FDG avid mediastinal nodes, D: Bilateral pulmonary nodules, E: Hepatosplenic hypodensities, F: Pelvic lymphadenopathy PET-CT: Positron emission tomography-computed tomography, F18-FDG: Fludeoxyglucose F18

A 40-year-old male presented with a history of fever associated with rigors and chills, cough, a loss of 5 Kg in weight, and increasing intensity of symptoms over three months before presentation. Diagnostic workup revealed extensive abdominal, mediastinal and hilar lymphadenopathy on computerized tomography (CT) scans. Biopsy of para-aortic lymph nodes showed cHL, nodular sclerosis type. He had stage IVB disease with an International Prognostic Score (IPS) of 5/7 based on staging a positron emission tomography (PET) scan that revealed hypermetabolic nodal disease above and below the diaphragm, hepatic, splenic, marrow, and pulmonary involvement (Figure 1). The patient also had a high erythrocyte sedimentation rate (ESR) of 65 mm/first hour, hemoglobin 8.4 g/dL, total leukocyte count 4860/ul, lymphocyte count 330/ul, and serum albumin 3.4 g/dL. Pre-chemotherapy echocardiogram was unremarkable. Viral serology was negative for human immunodeficiency virus (HIV), hepatitis B surface antigen (HBsAg), and antibody immunoglobulin G (IgG) and IgM in the hepatitis B core antibody (HBcAb) test. The hepatitis C virus (HCV) antibody was borderline reactive but HCV was not detected by polymerase chain reaction (PCR). An extensive workup was carried out to rule out the concomitant infectious causes before initiation of chemotherapy: serum procalcitonin (1.39 ng/ml), Covid-19 PCR (not detected), malarial antigen and parasite (negative by immunochromatographic test and blood film), serial blood and urine cultures (no growth identified). Chest radiograph revealed widened mediastinum representing bulky nodal disease and bilateral patchy lower lobe airspace changes. Expert opinion from infectious diseases and medical oncology specialists concluded that this fever was disease-related and that definitive treatment with chemotherapy should be started. He was offered doxorubicin, bleomycin, vinblastine, and dacarbazine (ABVD) regimen: Adriamycin (25 mg/m2), bleomycin (10mg/m2), vinblastine (6 mg/m2), and dacarbazine (375 mg/m2).

Due to persistent fever, he was admitted for the first cycle of chemotherapy, and was transfused packed red cells. The patient had an uneventful administration of Adriamycin, bleomycin, and vinblastine. However, 20 minutes after receiving bleomycin, he developed a dry cough that progressed to shortness of breath and high-grade fever. On examination, his heart rate was 160 beats per minute, blood pressure 200/110 mmHg, temperature 40°C, respiratory rate 30 per minute with an expiratory wheeze, and oxygen saturation 89% on room air needing 15 liters per minute of oxygen supplementation via non-rebreather mask. He was given hydrocortisone 100 mg and pheniramine 25 mg intravenously with no significant improvement in his symptoms. The intensive care team was consulted and considering anaphylaxis as a cause of his clinical deterioration, he was given 0.5 mg adrenaline 1:1000 solution intramuscularly with two repeated doses at five-minute intervals with no improvement in his condition. He was shifted to the intensive care unit (ICU) for further management where he was put on non-invasive positive pressure ventilation via bilevel positive airway pressure (BiPAP) and continuous intravenous adrenaline infusion. The chest X-ray posteroanterior (CXR-PA) view performed in the ICU revealed stable pulmonary findings including bilateral airspace disease and mild right pleural effusion since prior radiograph. Arterial blood gases (ABGs) revealed pH 7.29, PaCO2 45 mmHg, and PaO2 115 mmHg.

**Figure 2:**
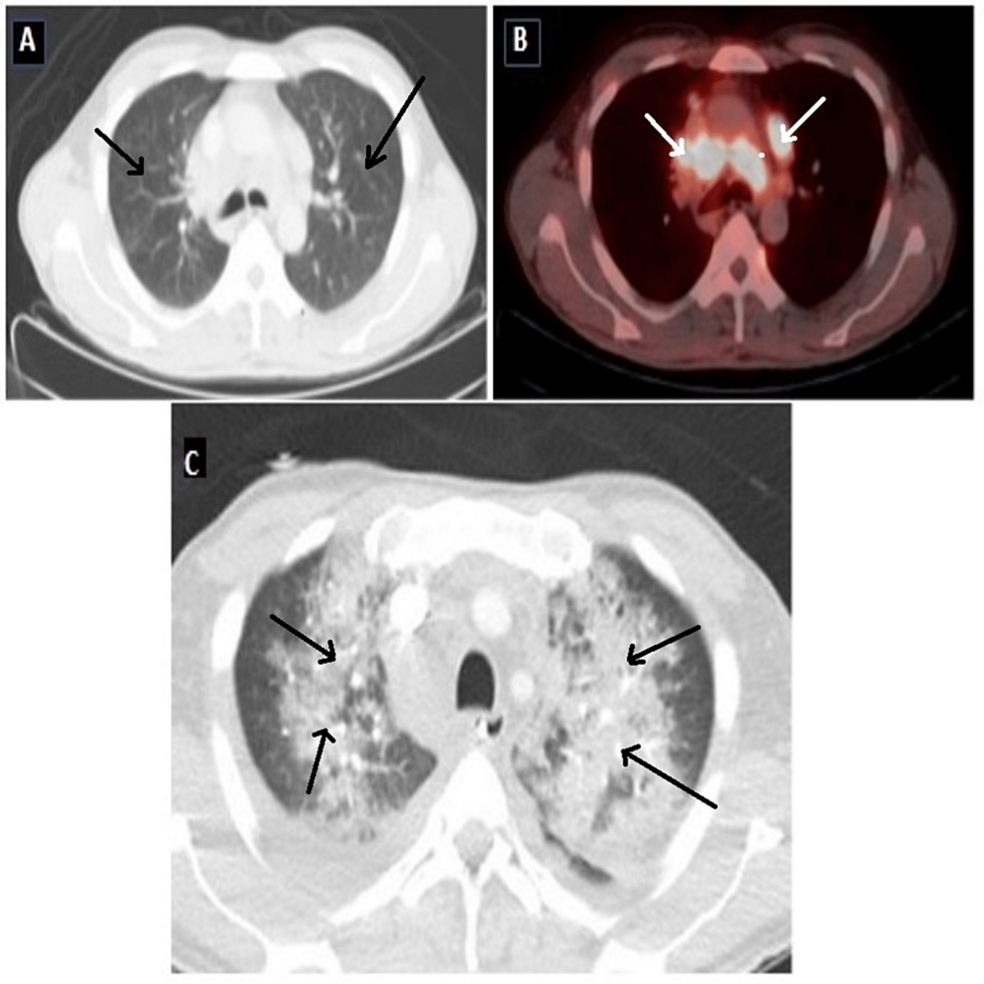
Baseline F18-FDG PET-CT scan Axial images of the CT (A) and Fusion PET-CT (B) at the level of tracheal bifurcation shows normal lung parenchyma and hypermetabolic mediastinal lymphadenopathy respectively. ​The CT scan (C) through the chest shows bilateral patchy lung infiltrates suggestive of bleomycin-induced lung injury. PET-CT: Positron emission tomography-computed tomography, F18-FDG: Fludeoxyglucose F18

Given his clinical condition and series of events, he was provisionally diagnosed by the on-call oncology team to have bleomycin-induced fulminant hyperpyrexia supported by a deranged coagulation profile with a high international normalized ratio (INR) of 1.52, increased activated partial thromboplastin time (43 seconds, normalized ration (NR) 22-31 seconds), increased D-Dimers (3.76 0.5 mg/L fibrinogen equivalent unit (FEU), NR <0.5), an elevated serum fibrinogen level (634 mg/dL), serum lactate (20 mg/dL), and C-reactive protein (CRP) (243.4 mg/L). Initial serum troponin level was high 0.607 ng/mL, and it rose to 1.373 ng/mL in the following six hours but it fell to 0.784 ng/mL a further six hours apart. The B-type natriuretic peptide (BNP) level was 537.5 pg/mL. The patient received a packed red blood cell transfusion as his hemoglobin levels also showed a sharp fall. He was managed with intravenous fluids, intravenous hydrocortisone 100 mg six hourly, and broad-spectrum antibiotics. His respiratory distress kept on deteriorating and he was electively intubated for mechanical ventilation. A CT pulmonary angiogram (Figure 2) was performed which ruled out pulmonary embolism, however, there were patchy infiltrates along with bilateral pleural effusion. 

A repeat echocardiogram revealed a subnormal left ventricular ejection fraction of 45% to 50%, a trivial pericardial effusion, and dilated inferior vena cava. An extensive workup was done to rule out infection/pneumonia as a cause of the patient's respiratory failure, including serial blood cultures, bronchoscopy, and bronchoalveolar lavage (BAL). All were negative for any evidence of infection and a pulmonology review ruled out any such possibility. He remained on a mechanical ventilator for 10 days, improved gradually, and was shifted to the general ward after successful extubation. Currently, he has completed chemotherapy with a complete metabolic response an the end-of-treatment PET-CT scan and is on surveillance.

Patient two

A 45-year-old male, newly diagnosed with cHL with a background of B symptoms and bulky mediastinal lymphadenopathy with impending airway compromise, was admitted with high-grade fevers and persistent vomiting. He was otherwise hemodynamically stable and was maintaining oxygen saturations on room air with no respiratory compromise. The initial extensive infectious screen was negative and the high-grade fevers were thought to be disease-related. He was admitted for emergent chemotherapy and received day one of the first cycle of ABVD chemotherapy. Shortly after completing bleomycin infusion, he developed a high fever of 40°C, heart rate of 140 per minute, shortness of breath with a respiratory rate of 40 per minute, and was unable to maintain saturations on room air with significant respiratory distress. He was managed with intravenous hydrocortisone, pheniramine, and 0.5 mg adrenaline 1:1000 solution intramuscularly. He was shifted to intensive care where he was intubated and ventilated. He remained hypotensive and very tachycardic despite vasopressor support. Unfortunately, he could not maintain blood pressure on maximum inotropic support, arrested, and could not survive failing resuscitative measures.

## Discussion

Both of our patients were clinically similar with respect to their background disease characteristics as well as their timing of development of fulminant hyperpyrexia. Both had persistent fevers before starting chemotherapy without any superimposed infectious etiology and both of them developed hyperpyrexia, tachycardia and respiratory compromise within half an hour of administration of bleomycin. Both were managed as anaphylactic reaction, and intubated subsequently, however, patient two suffered a cardiac arrest and passed away within 12 hours of bleomycin infusion.

Standard treatment of advanced stage cHL is with six cycles of ABVD or escalated bleomycin, etoposide, doxorubicin, cyclophosphamide, vincristine, procarbazine, prednisone (BEACOPP), especially in the case of patients younger than 60 years of age with a high IPS of >4/7 [16]. Bleomycin is an integral component of both of these regimens. More recently, brentuximab-vedotin in combination with adriamycin, vinblastine, and dacarbazine (AVD) (brentuximab vedotin (A)-AVD) has been advocated to be an alternate treatment regimen for advanced cHL with lesser incidence of pulmonary toxicity i.e., 10% with bleomycin as compared to <5% with brentuximab vedotin [17-18]. As detailed above, fever post-bleomycin injection is common, occurring in 50% to 60% of patients, and is dose-dependent [4]. Patients may develop fever within six hours of administration of bleomycin which is associated with chills and temperature may remain elevated for several hours. In <1% cases, patients develop high-grade fever, diaphoresis, respiratory distress needing oxygen supplementation and mechanical ventilation, and disseminated intravascular coagulation leading to death in its severe form. This has been labeled as BIFH and has been reported exclusively in patients with lymphoma with B symptoms, particularly with a history of high fevers. The exact mechanism of BIFH is uncertain, however, it is postulated to be due to an overwhelming toxic response to bleomycin superimposed on already established pyrexia as part of B symptoms of lymphoma [19]. Bleomycin has been shown to release endogenous pyrogens like tumor necrosis factor (TNF) and interleukin-1 (IL-1) and the release of these pyrogens is minimized by premedication with antihistamines, antipyretics, and steroids [7]. Another postulate of the underlying pathophysiology of this syndrome is excessive release of cytokines leading to hyperactivation of macrophages similar to cytokine release syndrome, however, the pathophysiology of this reaction is not well established [6]. A review of the literature suggests the association of this reaction with doses of bleomycin >25mg/m2 and reports suggest that this drug should not be used in patients with fever because such patients are supposed to be at increased risk of this complication [9]. In this perspective, the manufacturer's recommendation is to administer a test dose of bleomycin prior to administering its full dose to prevent this fulminant reaction; however, given the rarity of this syndrome, most institutions have abandoned this practice and monitor their patients for its potential side effects as the onset of reaction can occur anytime with any dose and test dose might not predict when the drug reaction may occur.

This complication is mainly seen in Hodgkin’s lymphoma cases. A common feature in both our cases was the presentation of high-grade fevers that worsened upon receiving the first cycle of ABVD. Hence, clinicians need to be cautious in such cases. Given the lethality of this syndrome, patients with advanced stage cHL can be treated with A-AVD instead to prevent such a lethal complication in addition to prospectively better treatment outcomes shown as per the ECHELON-1 trial [20].

## Conclusions

Classical Hodgkin’s lymphoma is a common clinical condition, usually treated with ABVD as a first-line regimen. Bleomycin is a safe and effective medicine with mild adverse reactions such as fever but leads to pulmonary toxicity in a fraction of patients leading to lifelong morbidity. A rare adverse reaction of bleomycin is fulminant hyperpyrexia which, although rare, is life-threatening toxicity. Fulminant hyperpyrexia has been seen mainly in patients with Hodgkin’s lymphoma and those with a preexisting history of high pyrexia leading to fatal events. The A-AVD is a safe and new leading option for patients with advanced-stage cHL allowing durable efficacy without the need for bleomycin as it lowers the toxicity while improving efficacy.
